# Comparison of plasma fatty acid binding protein 4 concentration in venous and capillary blood

**DOI:** 10.1371/journal.pone.0226374

**Published:** 2019-12-11

**Authors:** Shigeharu Numao, Yoshinori Nagasawa, Naomi Goromaru, Shunichi Tamaki

**Affiliations:** 1 Department of Sports and Life Sciences, National Institute of Fitness and Sports in Kanoya, Kagoshima, Japan; 2 Department of Health and Sports Sciences, Kyoto Pharmaceutical University, Kyoto, Japan; 3 Yamashina Takeda Racto Clinic, Kyoto, Japan; University of Dschang Faculty of Medicine and Pharmaceutical Sciences, CAMEROON

## Abstract

Circulating fatty acid binding protein 4 (FABP4) is associated with various diseases and simple and less invasive techniques for assessment of FABP4 concentration are required in clinical research setting. The purpose of the present study was to assess the correlation of plasma FABP4 concentration between venous and capillary blood in healthy young adults. Twenty-eight healthy young adults aged from 20 to 26 years (mean age, 22.2 ± 1.4 years, 14 males and 14 females) were included. Paired resting blood samples were taken from the cubital vein (venous) and fingertip (capillary) blood. Plasma FABP4 concentration in both blood was analyzed by enzyme-linked Immunosorbent assay. Plasma FABP4 concentration did not differ significantly between venous and capillary blood (−0.11± 0.75 ng/mL, *p* = 0.447, 95%CI: -0.402–0.182). Pearson’s correlation coefficient for plasma FABP4 concentration between venous and capillary blood samples suggests strong correlation (r = 0.961, *p* < 0.001). The Bland & Altman plot showed a non-significant bias (−0.11 ± 0.75 ng/mL, *p* = 0.684) and the 95% limits of agreement ranged from −1.59 to 1.37 ng/mL. FABP4 concentration in both venous and capillary blood was significantly higher in females than in males (venous blood: *p* = 0.041; capillary blood: *p* = 0.049). These results suggest that capillary blood sampling can detect gender difference and is useful for the assessment of FABP4 concentration.

## Introduction

Fatty acid binding proteins (FABPs) are a family of 14–15 kDa cytosolic lipid chaperones that regulate lipid trafficking and response in cells [1]. One of these members, fatty acid binding protein 4 (FABP4), also known as adipocyte FABP or adipose protein 2, is highly expressed in adipocytes and macrophages [2, 3, 4]. FABP4 is known to be secreted mainly from adipocytes [5, 6], and circulating FABP4 concentration has been reported to be associated with a risk of various diseases, such as atherosclerosis [7, 8], insulin resistance [9], type2 diabetes [10], hypertension [9, 11], dyslipidemia [9, 12], cardiovascular diseases [13,14], and cancer [15]. The potential treatment of metabolic disease by targeting circulating FABP4 concentration has been recently proposed [16]; therefore, there is increasing interest in circulating FABP4 concentration as a disease biomarker.

With rising curiosity in the association between circulating FABP4 concentration and various diseases in a clinical setting, researchers seek simple and less invasive techniques, reflecting profiles of FABP4 concentration close to the *in vivo* situation. Assessment of circulating FABP4 concentration usually requires collection of blood. Venous blood sampling, typically from the antecubital vein, has been widely used. However, a simpler method of blood collection is preferred in clinical research settings, since venous blood sampling is relatively invasive, requires a trained phlebotomist, generates biological waste, creates participant discomfort, and interrupts physical activity [17, 18]. Alternatively, capillary blood sampling from the fingertip has been used as a simplified method to obtain a blood sample. It is considered minimally invasive and can avoid excessively restricting activities and reduce the discomfort of participants. Therefore, capillary blood sampling is ideal in clinical research settings. Nevertheless, to the best of our knowledge, difference in circulating FABP4 concentration between venous and capillary blood has not yet been investigated. Depending on the blood component, circulating concentrations can greatly differ between venous and capillary blood [19, 20, 21]; therefore, it is important to study whether FABP4 concentration is the same when using capillary blood as an alternative blood sampling method.

As a pilot study, the present study assessed correlation of plasma FABP4 concentration between venous and capillary blood in healthy young adults. It was hypothesised that plasma FABP4 concentration in capillary blood could accurately and precisely reflect the concentration in venous blood.

## Materials and methods

### Participants

Twenty-eight healthy young adults aged from 20 to 26 years (mean age, 22.2 ± 1.4 years, 14 males and 14 females) participated in the present study. All participants were recruited from the undergraduate and graduate student populations using an ethics board-approved flyer. As metabolic, cardiovascular diseases and cancer effect circulating FABP4 concentration, major criteria for exclusion were the presence and history of metabolic [7, 8, 9, 10, 11, 12, 13, 14], cardiovascular diseases or cancer [15]. However, none of the participants were being medically treated or had a history of metabolic, cardiovascular diseases or cancer. All participants were non-smokers and did not take any medication or supplements. The purpose, design and risks of the present study were explained to all participants, and each provided written informed consent. The study conformed to the principles outlined in the Declaration of Helsinki and was approved by the ethics committee of Kyoto Pharmaceutical University (18–12).

### Study procedure

The participants visited the experimental place twice. On the first day, they visited our laboratory and completed measurement of anthropometry and body composition. Additionally, they were interviewed about health condition (current and previous disease history). On the second day, they visited the hospital for blood sample collection. Participants were instructed to refrain from exercise for at least 24 hours and from the consumption of food for at least 4 hours.

### Measurements of anthropometry and body composition

For each participant, height was measured to the nearest 0.1 cm using a stadiometer. Weight, fat mass (FM), fat-free mass (FFM) and skeletal muscle mass (SMM) were measured to the nearest 0.1 kg using the impedance method (InBody430; InBody Japan, Tokyo, Japan). Body mass index (BMI) was calculated as the weight in kilograms divided by the square of the height in metres.

### Blood sampling and analysis

After measuring blood pressure and resting for a 10-min period in the supine position, paired blood samples were taken by the hospital nurses from the cubital vein and the fingertip within a 5-minute difference. Venous blood samples were collected in a 2-mL tube containing heparin (VenojectII, TERUMO CORPORATION, Tokyo, Japan). Fingertip blood (capillary blood) samples were collected in two 150-uL microtubes containing heparin (‘Kantan’ tube, EIKEN CKEMICAL CORPORATION, Tokyo, Japan). After disinfecting the fingertip blood with alcohol, a single-use lancing device was used to puncture it. Approximately 300 μL of capillary blood was collected in the microtubes. The total duration of the procedure was approximately 2 min. Venous blood samples were centrifuged at 3000 × *g* for 10 min at 4°C within 30 min of collection; capillary blood samples were centrifuged at 2000 × *g* for 5 min at room temperature within 5 min of collection. Following centrifugation, the plasma obtained from each sample (plasma from venous and capillary blood: approximately 1 ml and 150 μL, respectively), was transferred to plastic tubes and immediately stored at −40°C until further analysis.

Plasma FABP4 concentration was analysed using an enzyme-linked immunosorbent assay (ELISA) (R&D system Inc., Minneapolis, the United State of America; intra-assay CV: < 5.8%). The total volume of the required plasma of each sample was 20 μL, and the ELISA procedure was performed according to the manufacturer’s instructions [Quantikine ELISA Human FABP4 Immunoassay (R&D system Inc.)]. To eliminate inter-assay variation, samples from each participant were analysed in the same run.

### Statistical analysis

All data are represented as the mean ± standard deviation (SD) and were tested for normality using the Kolmogorov–Smirnov test; sample size was calculated to detect a large effect (Cohen’s *d* = 0.80 and *r* = 0.50). A sample size of 15–26 would be required to have approximately 80% power to detect a large effect at 0.05 significance. A paired *t*-test was used to compare differences in plasma FABP4 concentration between venous and capillary blood. Bland and Altman plot with 95% limits of agreement (± 1.96 x SD) [22] and Pearson’s correlation coefficient were used to assess the correlation between the venous and capillary blood samples. Regression analysis, with the difference in plasma FABP4 concentration between the venous and capillary blood samples as the dependent variable and the mean between venous and capillary plasma FABP4 concentration as the independent variable, was used to evaluate proportional bias in a Bland and Altman plot. An unpaired *t*-test was used to compare differences in physical characteristics between males and females. Moreover, analysis of covariance (ANCOVA) with % fat and FFM as covariates was used to compare differences in physical characteristics between males and females. The effect size (ES) was calculated using Cohen’s *d* (small: ≥ 0.20, medium: ≥ 0.50, or large: ≥ 0.80) for comparison of physical characteristics and plasma FABP4 concentration in venous and capillary blood. Statistical analyses were performed using the SPSS version 24 software (IBM Corporation, NY, USA). Statistical significance was set at *p* < 0.05.

## Results

### Concordance of plasma FABP4 concentration between venous and capillary blood

**Fig 1:**
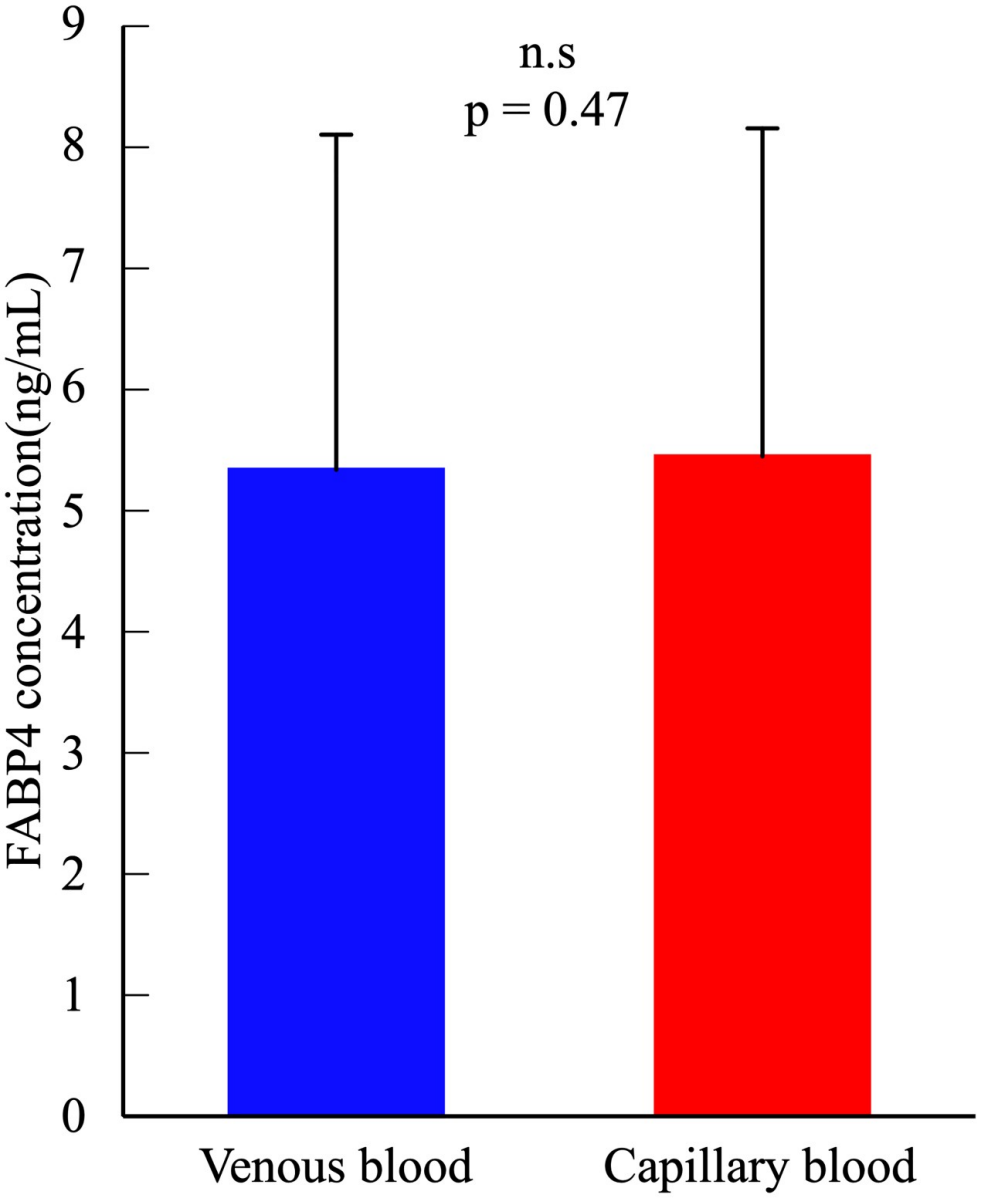
Difference in plasma FABP4 concentration between venous and capillary blood. Data are presented as the mean ± SD. There was no significant difference in plasma FABP4 concentration between venous and capillary blood (*p* = 0.447).

**Fig 2:**
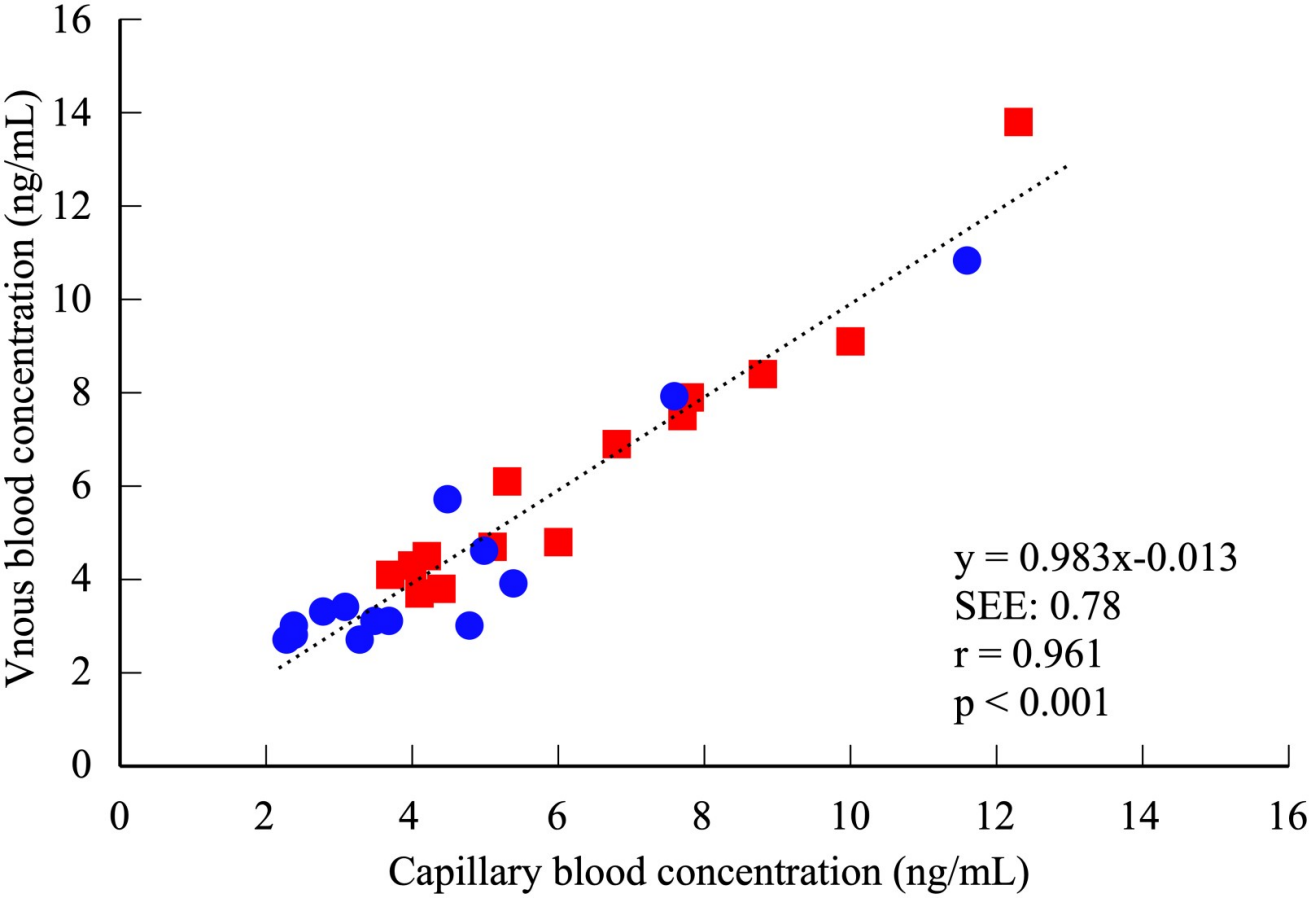
Correlation of plasma FABP4 concentration between venous and capillary blood. Circle and triangle plots represent males and females, respectively. Pearson’s correlation coefficients, All participants: r = 0.962 (*p* < 0.001), males: r = 0.945 (*p* < 0.001), females: r = 0.970 (*p* < 0.001).

**Fig 3:**
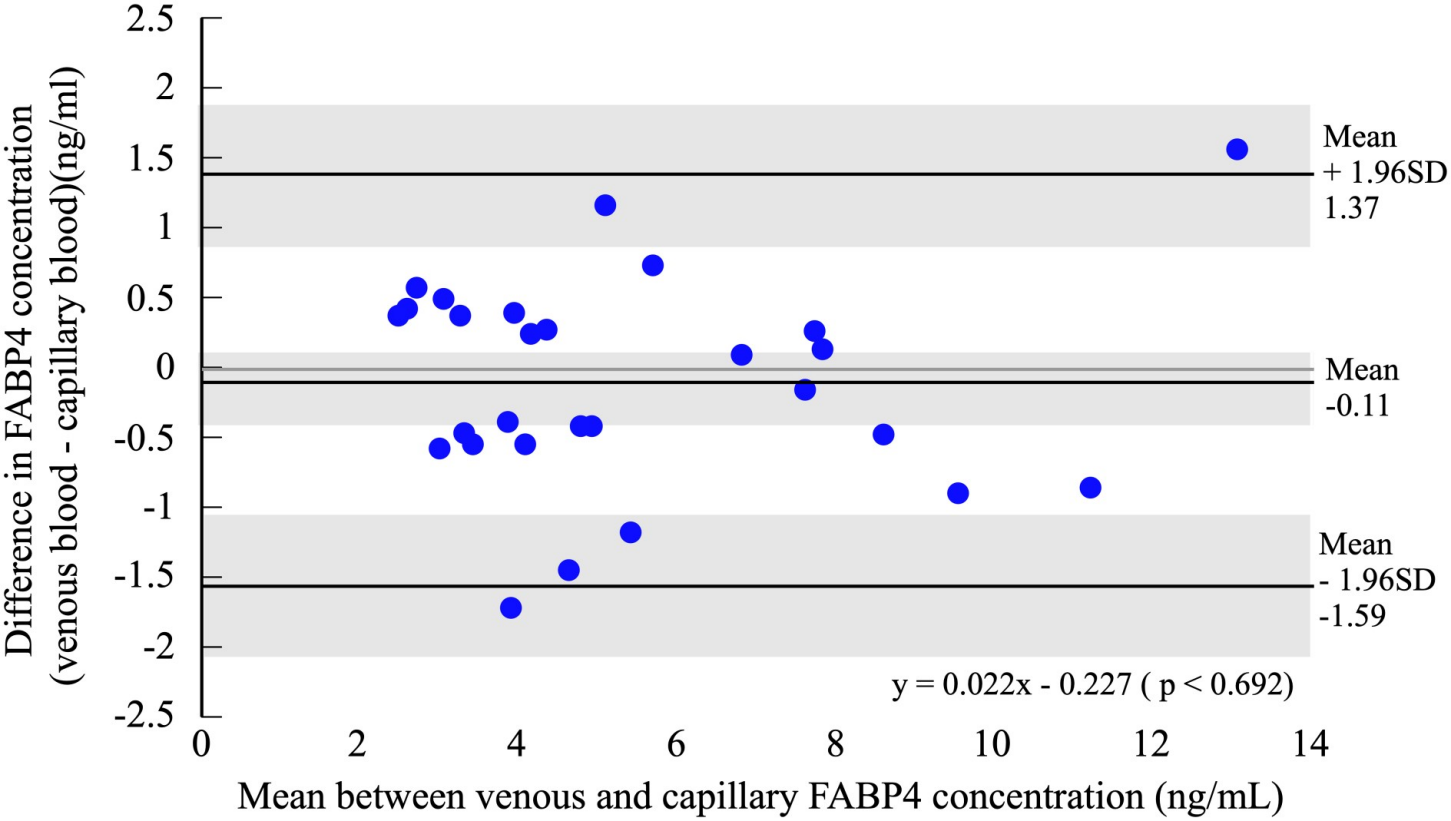
Bland and Altman plots showing the difference against the average plasma FABP4 concentrations between venous and capillary blood. Solid lines represent the mean bias and the 95% limits of agreement. Shadows are confidence intervals. There was no correlation between the difference and average plasma FABP4 concentrations between venous and capillary blood.

Plasma FABP4 concentration did not significantly differ between venous (5.34 ± 2.77 ng/ml) and capillary blood (5.45 ± 2.71 ng/ml) (t = -0.773, df = 27, *p* = 0.447, 95%CI: -0.402–0.182) (Fig 1). Pearson’s correlation coefficient in all participants and each gender for plasma FABP4 concentrations between venous and capillary blood was significantly high (All: r = 0.962, *p* < 0.001, males: r = 0.945, *p* < 0.001, females: r = 0.970, *p* < 0.001) (Fig 2). The Bland and Altman plot showed a non-significant bias in all participants (-0.11 ± 0.75 ng/mL, t = -0.773, df = 27, *p* = 0.447, 95%CI: -0.402–0.182) in plasma FABP4 concentrations between venous and capillary blood (Fig 3). Regression analysis in all participants showed that the estimated regression equation was not significant (y = 0.022x–0.227, *p* = 0.692). The 95% limits of agreement ranged from −1.59 to 1.37 ng/mL. Two outliers were observed in Bland & Altman plot, but 92.8% plots were included in the 95% limits of agreement. In each gender, the Bland and Altman plot showed a non-significant bias and regression analysis showed that the estimated regression equation was not significant (S1 Fig).

### Difference in plasma FABP4 concentration by gender

Age and BMI did not significantly differ between males and females (Table 1), but there was a significant difference in height, weight, % fat, FM, FFM and SMM (all *p* < 0.001). Plasma FABP4 concentration in both venous and capillary blood was significantly higher in females than in males (venous blood: t = -2.153, df = 26, *p* = 0.041, 95%CI: -4.13 –-0.10, *d* = 0.80; capillary blood: t = -2.056, df = 26, *p* = 0.049, 95%CI: -3.98 –-0.0009, *d* = 0.77). These differences were not significant following adjustment for % fat and FM.

**Table 1:** Physical characteristics of all participants.

	All	Male	Female	*p*-value	ES
Sample size	28	14	14		
Age (yrs)	22.1 ± 1.4	22.5 ± 1.7	21.8 ± 1.0	0.140	0.57
Height (cm)	165.7 ± 7.4	171.7 ± 4.1	159.6 ± 4.3*	<0.001	2.88
Weight (kg)	56.8 ± 7.5	61.8 ± 6.5	51.7 ± 4.6*	<0.001	1.79
BMI	20.6 ± 2.0	21.0 ± 2.3	20.3 ± 1.6	0.378	0.35
% Fat (%)	19.9 ± 7.7	14.1 ± 5.3	25.7 ± 4.9*	<0.001	2.27
FM (kg)	11.1 ± 4.0	8.8 ± 3.5	13.4 ± 3.2*	<0.001	1.37
FFM (kg)	45.7 ± 8.8	53.0 ± 5.9	38.3 ± 2.9*	<0.001	3.16
SMM (kg)	25.3 ± 5.5	29.9 ± 3.7	20.7 ± 1.8*	<0.001	3.16
Venous FABP4 concentration (ng/mL)	5.34 ± 2.77	4.28 ± 2.36	6.39 ± 2.82*	0.041	0.81
Capillary FABP4 concentration (ng/mL)	5.45 ± 2.71	4.45 ± 2.53	6.44 ± 2.59*	0.049	0.77

All data are shown as the mean ± SD. BMI, body mass index; FM, fat mass; FFM, fat-free mass; SMM, skeletal muscle mass; FABP4, fatty acid binding protein 4; ES, effect size (Cohen’s *d*). *p*-value: unpaired *t*-test between males and females.

*p < 0.05 vs male.

## Discussion

The present study is the first to compare plasma FABP4 concentrations between venous and capillary blood in healthy adults. Our data demonstrate that plasma FABP4 concentration in capillary blood was almost identical to that in venous blood. Additionally, plasma FABP4 concentration in both venous and capillary blood showed higher values in females than in males. These findings suggest that capillary blood samples can detect a gender difference in circulating FABP4 concentration and are fully interchangeable with venous samples for assessing circulating FABP4 concentration.

As no previous studies have reported a difference in circulating FABP4 concentrations between venous and capillary blood, whether circulating FABP4 concentration in capillary blood corresponds to that in venous blood was unclear. Therefore, we assessed the demonstrative reliability of plasma FABP4 concentration in venous and capillary blood using several statistical methods. Firstly, a paired *t*-test was adopted to assess systematic bias. The statistical result showed no significant difference in plasma FABP4 concentration between venous and capillary blood, suggesting that systemic bias of plasma FABP4 concentration in capillary blood is small as compared with that in venous blood. Secondly, correlation analysis was used to assess relative reliability. Pearson’s correlation coefficient was very high (0.961) and statistically significant, suggesting that relative reliability of plasma FABP4 concentration in capillary blood is sufficient enough when compared to that in venous blood. Finally, the Bland & Altman plot [22] was utilized to assess absolute reliability. The Bland & Altman plot showed that bias was -0.11, and 94% of the plot was included within the 95% limits of agreement ranging from −1.58 to 1.36 ng/mL, suggesting that plasma FABP4 concentration in capillary blood had a smaller bias and an agreement with that in venous blood. On the other hand, the 95% limits of agreement must be also interpreted from a clinical perspective. Although the reference value of circulating FABP4 concentration has not yet been defined, several studies have reported that difference in circulating FABP4 concentrations by the presence or absence of disease. Serum FABP4 concentration in type 2 diabetic patients (approximately 55 ng/mL) is higher than that in non-type 2 diabetic patients (approximately 20 ng/mL) [10]. Essential hypertensives (approximately 22 ng/mL) have a higher serum FABP4 concentration than normotensives (approximately 15 ng/mL) [11]. Additionally, serum FABP4 concentrations in population with metabolic syndrome (male: 29.34 ng/mL, female: 37.00 ng/mL) is significantly higher than that in those without metabolic syndrome (male: 16.63 ng/mL, female: 17.81 ng/mL) [9]. Although difference in blood sample analysis method should be taken into accounts, relatively great difference (7 to 25 ng/mL) in circulating FABP4 concentration leads to detection of several diseases. Based on these studies, the range of 95% limits of agreement in the present study is narrow (approximately ±1.5 ng/mL) and capillary blood sample is acceptable for the assessment of circulating FABP4 concentration in clinical research. These statistical data and clinical interpretation suggest that the use of capillary blood samples can be interchangeable with venous blood samples. It is likely that the strong association between venous and capillary FABP4 concentration is because the balance between “production and/or secretion” and “absorption and/or clearance” of circulating FABP4 in peripheral blood as stable as it is in venous blood.

Plasma FABP4 concentration was significantly higher in females than in males in the present study. This finding is consistent with previous studies [7, 8, 9, 12, 23]. FABP4 is expressed and secreted from adipocytes [5, 6], and the amount of body fat is generally higher in females than in males. One of the reasons for the gender difference may be the amount of body fat. Previous studies have reported that serum FABP4 concentrations were correlated with BMI [9, 23], but our results showed that plasma FABP4 concentrations in both venous and capillary blood did not correlate with BMI. However, there was a correlation with % fat and FM (S1 Table) and the significant difference in plasma FABP4 concentration disappeared after adjustment for % fat or FM. These findings suggest that the amount of body fat directly contributes to the gender difference in circulating FABP4 concentration. Furthermore, the relative narrow range of BMI values in our sample (16.3–23.9) as compared with those in previous studies may account for the different results.

Increased circulating FABP4 concentration can predict a risk of various diseases [7, 8, 9, 10, 11, 12, 13, 14]. Although exercise reduces a risk of various diseases [24], exercise may increase circulating FABP4 concentration. Several lines of evidence have been reported in animals and humans. Ten-week aerobic training and 4-week resistance training have been shown to increase gene expression of adipocyte FABP4 protein and plasma FABP4 concentration in rats [25, 26]. In humans, endurance-trained individuals have a much higher FABP4 mRNA and protein expression level in skeletal muscle as compared with control individuals [27]. In addition, acute high-intensity exercise transiently elevates serum FABP4 concentration [28]. Thus, the response in circulating FABP4 concentration to exercise appears to be paradoxical. Nevertheless, the evidence of association between circulating FABP4 concentration and exercise is limited. Further studies are required to adequately determine the effect of exercise on circulating FABP4 concentration. In those studies, our findings can be utilized. Our capillary sampling can be more useful to assess circulating FABP4 concentration, because it is not required to interrupt physical activity.

The present study has several limitations. Firstly, the participants were young healthy males and females; therefore, our findings may not be applicable to obese individuals, older adults with or without diseases and cancer patients. It is possible that FABP4 concentration would be higher in patients with metabolic or cardiovascular diseases than in young healthy individuals [7, 8, 9, 10, 11, 12, 13, 14] Moreover, cancer patients would have a greater circulating FABP4 concentration [15]. When considering potential selection bias, it is necessary to consider the correlation of circulating FABP4 concentration between venous and capillary blood in a population with higher circulating FABP4 concentration. Secondly, sampling of venous and capillary blood was performed under steady state conditions. Since metabolite concentration may fluctuate between venous and capillary blood under dynamic conditions (i.e., immediately after food consumption) [17, 29], our findings may not be applicable in the dynamic state. Thus, future studies in different populations and situations are warranted to widely generalize our results.

In conclusion, the findings of the present study demonstrate that capillary blood sampling is a reliable alternative to venous blood sampling to assess plasma FABP4 concentration in healthy young adults. Capillary blood sampling will be a useful method in clinical research settings.

## Supporting information

S1 Table Pearson’s correlation coefficients between plasma FABP4 concentration and physical characteristics. (DOCX)

S1 Fig Bland and Altman plots showing the difference against the average plasma FABP4 concentrations between venous and capillary blood in males and females. Solid lines represent the mean bias and the 95% limits of agreement. Shadows are confidence intervals. There was no correlation between the difference and average plasma FABP4 concentrations between venous and capillary blood.
(TIF)
