# Supplementary material for: Comparison of plasma fatty acid binding protein 4 concentration in venous and capillary blood
Source: PLoS One. 2019 Dec 11;14(12):e0226374. doi: 10.1371/journal.pone.0226374 (PMC6905543; doi:10.1371/journal.pone.0226374)
Supplement: S1 Table — (DOCX) [file pone.0226374.s001.docx]

**S1 Table. Pearson’s correlation coefficients between plasma FABP4 concentration and physical characteristics.**

|  | | Age | Weight | BMI | % Fat | FM | FFM | SMM |
| --- | --- | --- | --- | --- | --- | --- | --- | --- |
| Venous FABP4 concentration | r | -0.243 | -0.160 | 0.171 | 0.442 * | 0.456 * | -0.348 | -0.340 |
|  | *p* | 0.213 | 0.415 | 0.386 | 0.019 | 0.015 | 0.069 | 0.076 |
| Capillary FABP4 concentration | r | -0.216 | -0.120 | 0.198 | 0.416 * | 0.444 * | -0.308 | -0.304 |
|  | *p* | 0.269 | 0.542 | 0.313 | 0.028 | 0.018 | 0.110 | 0.115 |

BMI, body mass index; FM, fat mass; FFM, fat-free mass; SMM, skeletal muscle mass; FABP4, fatty acid binding protein 4. **p* < 0.05.

Plasma FABP4 concentration in both venous and capillary blood was significantly correlated with % fat, but was not significantly correlated with age, weight, BMI, FFM, or SMM.
