# Supplementary figures and images for: Comparison of plasma fatty acid binding protein 4 concentration in venous and capillary blood
Source: PLoS One. 2019 Dec 11;14(12):e0226374. doi: 10.1371/journal.pone.0226374 (PMC6905543; doi:10.1371/journal.pone.0226374)

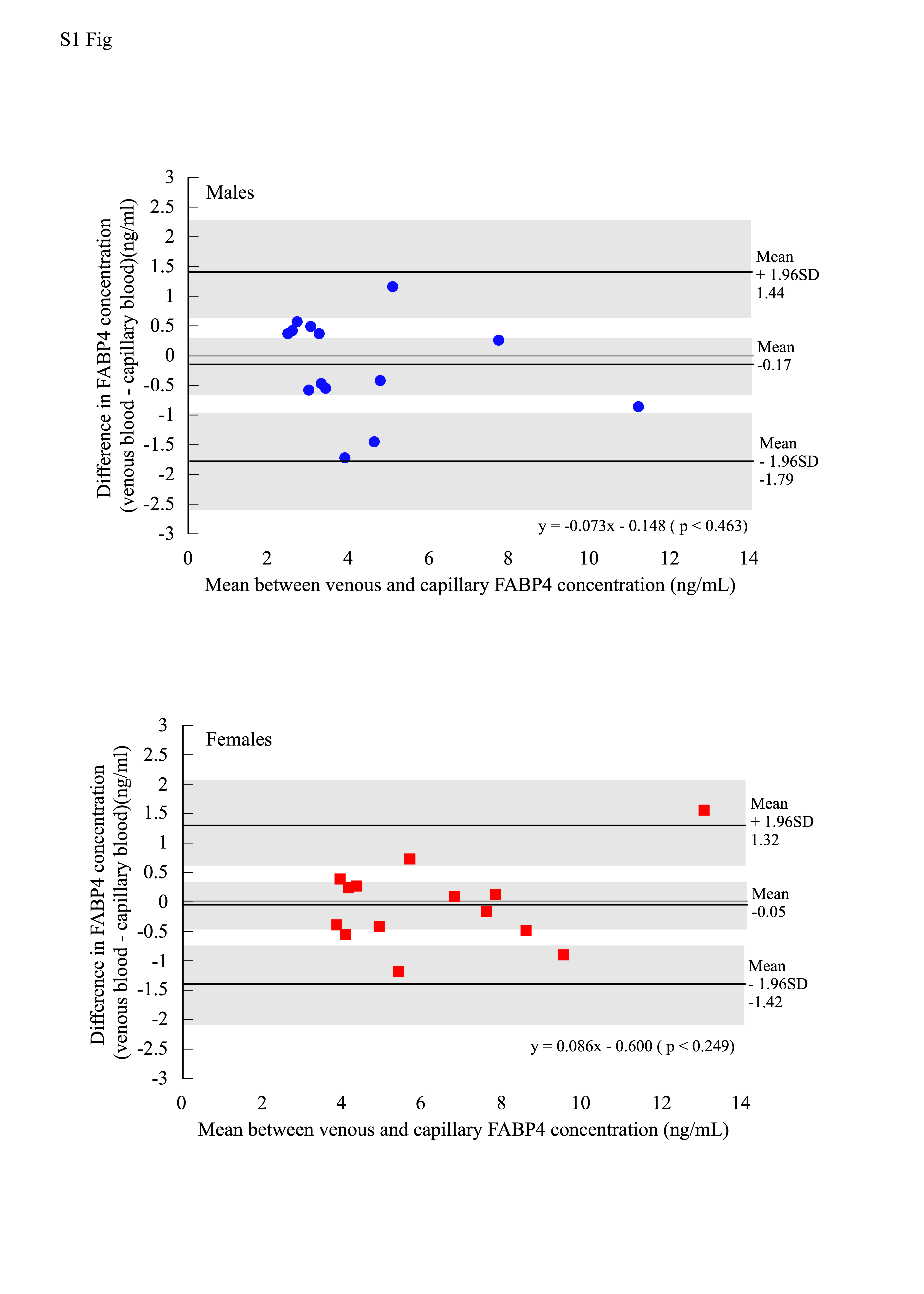

Supplement: S1 Fig — Solid lines represent the mean bias and the 95% limits of agreement. Shadows are confidence intervals. There was no correlation between the difference and average plasma FABP4 concentrations between venous and capillary blood. (TIF) [file pone.0226374.s002.tif]
